# Rab11FIP proteins link endocytic recycling vesicles for cytoskeletal transport and tethering

**DOI:** 10.1042/BSR20182219

**Published:** 2019-01-30

**Authors:** Laura M. Machesky

**Affiliations:** CRUK Beatson Institute and Institute of Cancer Sciences, University of Glasgow, Garscube Estate, Switchback Road, Bearsden, Glasgow, G61 1BD, U.K.

**Keywords:** cell migration, endocytic trafficking, endocytosis, integrins, rab gtpases

## Abstract

Regulated trafficking of internalised integrins and growth factor receptors enables polarisation of morphology and motility and enables lumen formation in multicellular structures. Recycling vesicles marked with Rab11 direct internalised cargo back to the plasma membrane to affect biological processes such as polarised trafficking and cancer cell invasion. A recent study by Ji and colleagues, provides insight into how the trafficking protein Rab11FIP2 links with the actin-based motor myo5b and the small GTPase Rab11 to regulate vesicle tethering and transport along actin filaments [1]. The authors used biochemical methods to demonstrate that Rab11a binds directly to the tail of myo5b and that Rab11FIP2 also forms direct interactions with both Rab11a and myo5b tails. These proteins essentially compete for binding to similar regions and thus can regulate the association and activity of each other. Ji and colleagues further demonstrate that Rab11a activates myo5b by binding to its globular tail and relieving a head-tail autoinhibition. Due to differing affinities between Rab11 and myo5b or Rab11FIP2, they propose that Rab11FIP2 mediates the association of myo5b with cargo vesicles, while Rab11a regulates the motor activity of myo5b. The present study thus elucidates how myo5b is regulated by its interactions with Rab11a and Rab11FIP2 and proposes a model for coordination of recycling vesicle tethering and motor activity. The present study has implications for how cells control polarity and motility in health and disease and suggests how Rab11FIP proteins might control motor protein activity and engagement for transport.

Extracellular signals and integrin engagement trigger internalisation and recycling of membrane-bound receptors and integrins through various pathways, depending on the context and cell type. Cells vary their trafficking of adhesion and signalling receptors through fast and slow recycling pathways and target some proportion of internalised membrane proteins for lysosomal degradation. The small GTPase Rab11 marks the major perinuclear endocytic recycling compartment that receives and transfers cargo such as growth factor receptors and integrins for transport back to the plasma membrane. The Rab11 recycling compartment has been called the slow recycling loop, as a faster route exists for vesicles that internalise through a Rab4 compartment and return to the plasma membrane without entering the Rab11 compartment [2]. The five-protein family of Rab11FIP proteins link endocytic vesicles with cytoskeletal motors for transport along actin and microtubules and regulate the balance of transport on these two filament types, to control cell motility, mitosis and polarity.

Rab11FIPs comprise a family of five proteins in mammals (Figure 1), all of which interact with Rab11 and homodimerise via a conserved C-terminal coiled-coil structure termed the Rab11 binding domain (RBD) (Figure 1). This domain can also mediate interaction with related Rabs, including Rab27a/b, which are implicated in polarised epithelial trafficking. Rab11FIP1, 2 and 5 contain an N-terminal C2 domain and are defined as Class I FIPs. The C2 domain mediates association with membranes through lipid binding. C2 domains generally mediate plasma membrane association, but in the case of Rab11FIP proteins, their primary association is with internalised endocytic vesicles. Rab11FIP1, also known as Rab coupling protein (RCP) targets to vesicles trafficking through invasive pseudopodia of cancer cells via interaction with phosphatidic acid generated by diacylglycerol kinase α [3,4]. Rab11FIP5 (RIP11) targets to neutral phospholipids (phosphatidylcholine/phosphatidylethanolamine) via its C2 domain in a magnesium ion-dependent manner [5], but the general mechanism of how the various Rab11FIP C2 domains interact selectively with lipids is surprisingly unknown. The class II RabFIPs (RabFIP3 and 4) do not have C2 domains and both contain an Arf GTPase binding site (Figure 1). Rab11FIP3 (ARFO1) contains a calcium-binding helix-loop-helix motif called the EF-hand (Figure 1), as well as an ARF binding sequence (ABD). Like its relative Rab11FIP4, it can connect to both Rab11 and ARF GTPases and thus act as a sensor of multiple inputs. Rab11FIP4 has no special domain at the N-terminus.

**Figure 1:**
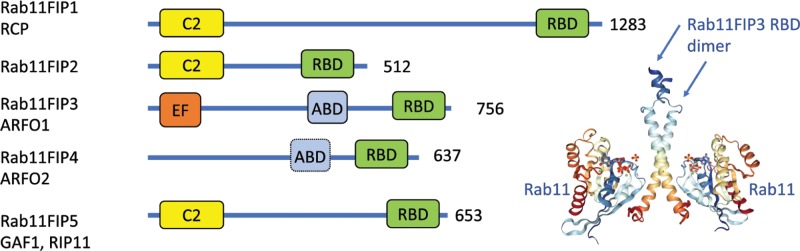
The Rab11FIP family of proteins Proteins are shown with domains represented as boxes. C2 (yellow) is C2 structural domain, RBD (green) is Rab11 binding domain, EF (orange) is EF-hand domain, ABD (light blue) is Arf GTPase binding domain, numbers represent amino acid number. Structure shows Rab11FIP3 RBD dimer in complex with two molecules of Rab11.

Rab11FIPs link recycling endocytic vesicles for tethering to or transport along actin filaments to establish and maintain cell polarity, build polarised structures or distribute cargo during cytokinesis (Figure 2). In particular, Rab11FIP2 interacts with Myo5b, a plus-end directed actin motor known to traffic synaptic vesicles and microvillus cargo [6–11]. Myo5b is a two-headed processive myosin motor and was recently characterised as a highly mechanosensitive myosin whose velocity and run length depend on the load or drag force experienced as a result of the cargo [7]. Myo5b is stimulated to assume an open active conformation when calcium and calmodulin bind, stimulating an interaction with actin filaments. However, excess calcium can cause dissociation [7], suggesting that calcium spikes in neuronal signalling may negatively regulate this complex. Ji and colleagues [1] revealed key mechanistic insights into how Rab11FIP2 and Rab11 can coordinate the interaction of Myo5b with membrane vesicles and regulate the activity of Myo5b as a motor. Rab11FIP2 acts as a bridge, binding both to Rab11 and Myo5b, and Rab11 acts more like a switch, causing activation of Myo5b, but in competition with Rab11FIP2 binding to Myo5b.

**Figure 2:**
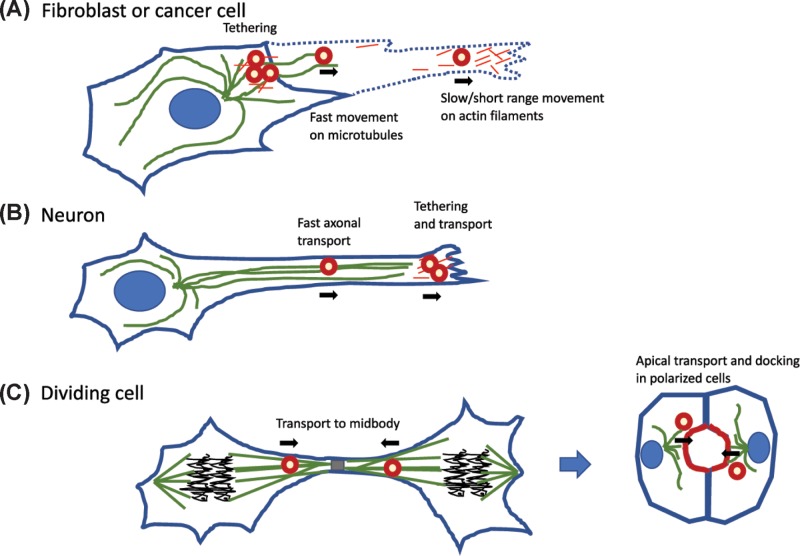
Schematic of Rab11FIP regulation of trafficking in various cell contexts (**A**) Fibroblastic or cancer cells show Rab11FIP vesicles (red circles) tethered with actin (red lines) and myo5a, moving along microtubules (green) or tethered/moving along actin filaments carrying cargo such as integrins and growth factor receptors out in the tips of invasive pseudopods. (**B**) Neuronal cells use Rab11FIP to regulate transport along the axonal microtubules and at the tips of axonal growth cones in interaction with actin filaments. (**C**) Dividing cells use Rab11FIPs to control transport of polarity proteins along microtubules toward the midbody and this transport results in eventual lumen formation after division is complete and as polarised cysts form.

There has been much discussion around whether Myo5b and actin filaments serve to actively transport recycling vesicles through the cell cortex or to tether them near the cell body. Evidence from multiple studies support both functions and suggests a dual role. For example, knockdown of Rab11FIP2 increased the motility of Rab11 vesicles in HeLa cells, suggesting that tethering was released [11]. The cargo Langerin, a lectin involved in antigen processing in dendritic cells, is tethered at the Rab11a-positive compartment together with Rab11FIP2 and myo5b and also associates with these molecules during late stage docking and release at the plasma membrane [8]. Tethered vesicles may thus provide a pool of primed cargo, ready to release in response to a signal.

Rab11FIPs also can interact with kinesins or dyneins for vesicle transport on microtubules. For example, Rab11FIP5 (RIP11) links directly to kinesin II (a complex of Kif3a, Kif3b and KAP3), a microtubule plus-end directed motor. In interphase cells, this complex is thought to direct internalised receptors through the perinuclear recycling endosome [12]. During mitosis, Rab11FIP5 is important for apical trafficking along central spindle microtubules toward the midbody to establish the new lumen of 3D MDCK cell cultured cysts [13]. It is not clear which (if any) kinesins link to other Rab11FIP proteins or which sequence motifs on Rab11FIPs mediate this interaction. Knockdown of Kif3a in interphase 2D cultured cells revealed accumulation of Rab11FIP5 vesicles in a peripheral actin-rich compartment near the plasma membrane [12], suggesting that kinesin may mediate dissociation of recycling vesicles from the actin cytoskeleton and promote the transition to transport along microtubules. Rab11FIP3 can also recruit dynein to Rab11a vesicles to mediate expulsion of pathogenic bacteria from infected bladder epithelial cells [9]. The exocyst complex is implicated in this vesicle-mediated expulsion [9] and suggests coordinated exocytosis of cargo can be regulated by Rab11FIP linkers in various contexts. Thus, vesicles can be tethered by myo5b on actin for rapid mobilisation in several contexts, allowing the cell to provide specific responsive delivery of cargo to regulate its activities.

The well-studied cargoes of Rab11FIP-containing vesicles include integrins, polarity proteins and tyrosine kinase receptors (recently reviewed in [2]). It is not intuitive why cells would need a ‘long loop’ for recycling, but Rab11FIP proteins and the Rab11 compartment allow coordination of specific membrane receptor ‘packages’ such as EGFR, ERBB2, Met, etc., to coordinate activation of signalling pathways with specific integrins (such as α5β1) [14]. These packages deliver into invasive pseudopods or to the tips of neuronal axons [2,3,15,16] to promote signalling together with adhesion and motility. A similar sorting mechanism may be required during mitosis, when vesicles traffic along spindle microtubules toward the midbody. These vesicles contain Rab11FIP5 and carry cargo, such as the apical specifying protein podocalyxin, to facilitate lumen formation [13]. Rab11FIP1 has also been implicated in trafficking of the dynamin-related protein Drp1 to mitochondria [17]. Drp1 can induce fragmentation of mitochondria and metabolic changes in a src kinase-dependent manner [17]. The Niemann–Pick receptor NPC1L1, named for the lipid storage disease, regulates the uptake of cholesterol by hepatocytes. Normally, the receptor is tethered by interaction with Rab11FIP2 and Myo5b and then released to the cell surface to boost uptake of cholesterol and facilitate lipid metabolic needs [6]. The receptor in complex with cholesterol can serve as an internal store of cholesterol in the endocytic recycling compartment of the cell. Thus, ternary complexes of RabFIPs such as myo5b-Rab11-Rab11FIP2 can act as a storage or holding reservoir to allow the cell to rapidly respond to a need for their release for signalling or other purposes such as lipid metabolism.

Due to their important roles in cargo sorting and trafficking, Rab11FIPs are also implicated in cancer, where alterations in trafficking can drive changes in invasive capacity, signalling and cell–cell communication potential. Rab11FIP1 (RCP) has been highlighted as a target of mutant p53 in various cancer types and its up-regulation promotes trafficking of integrins and other pro-invasive cargo [3,16]. Interestingly, RCP expression can alter the microenvironment around the tumour cells, such as affecting matrix deposition and exosome release [3] and thus drive the invasive potential of not only cancer cells but the stroma as well. RCP was also shown to promote expression of epithelial-to-mesenchymal transition, via the transcription factor Slug, in breast and ovarian cancer cells *in vitro* [18]. Other Rab11FIPs have also been implicated in cancer cell invasion and migration or proliferation *in vitro*, but the connection with human cancer is not yet clear. Rab11FIP1 and Rab11FIP4 were both correlated with poor survival in pancreatic cancer and shown to be important for proliferation and metastasis [19,20]. Rab11FIPs have emerged as prognostic indicators in ovarian cancer and an unbiased screen for novel splice variants revealed an isoform Rab11FIP4 as specifically expressed in ovarian tumours but not normal ovary or fallopian tube [21,22].

Rab11FIP proteins play an interesting role coordinating vesicle interactions with myosin and kinesins or dyneins, to promote movement along actin or microtubules or tethering to the actin cytoskeleton. Ji and colleagues [1] have shed light on the molecular interactions between myo5b, Rab11FIP2 and Rab11a, revealing how Rab11FIP2 coordinates not only formation of this ternary complex but activation of myo5b motor activity. Further studies of the biological relevance of this and other interactions of the five Rab11FIP proteins will reveal how these adapters control polarised trafficking of multiple cargo and delivery of specific ‘packages’ of activated receptors and adhesion molecules to regulate cell migration, division and signalling.

## Abbreviations

ARF: ADP ribosylation factor
EF: EF-Hand, a protein structural domain that binds to calcium
RBD: Rab11 binding domain
RCP: Rab coupling protein
SRC: A tyrosine kinase originally identified in Rous Sarcoma Virus
